# Adipose Tissue Dysfunction and the Role of Adipocyte-Derived Extracellular Vesicles in Obesity and Metabolic Syndrome

**DOI:** 10.1210/jendso/bvae126

**Published:** 2024-06-25

**Authors:** Alejandra Sandoval-Bórquez, Pablo Carrión, María Paz Hernández, Jorge A Pérez, Alejandra Tapia-Castillo, Andrea Vecchiola, Carlos E Fardella, Cristian A Carvajal

**Affiliations:** School of Medical Technology, Faculty of Science, Pontificia Universidad Católica de Valparaiso, Valparaiso 2373223, Chile; Center for Translational Research in Endocrinology (CETREN-UC), Pontificia Universidad Católica de Chile, Santiago 8330074, Chile; Department of Endocrinology, School of Medicine, Pontificia Universidad Católica de Chile, Santiago 8330077, Chile; Center for Translational Research in Endocrinology (CETREN-UC), Pontificia Universidad Católica de Chile, Santiago 8330074, Chile; Department of Endocrinology, School of Medicine, Pontificia Universidad Católica de Chile, Santiago 8330077, Chile; Center for Translational Research in Endocrinology (CETREN-UC), Pontificia Universidad Católica de Chile, Santiago 8330074, Chile; Department of Endocrinology, School of Medicine, Pontificia Universidad Católica de Chile, Santiago 8330077, Chile; Center for Translational Research in Endocrinology (CETREN-UC), Pontificia Universidad Católica de Chile, Santiago 8330074, Chile; Department of Endocrinology, School of Medicine, Pontificia Universidad Católica de Chile, Santiago 8330077, Chile; Center for Translational Research in Endocrinology (CETREN-UC), Pontificia Universidad Católica de Chile, Santiago 8330074, Chile; Department of Endocrinology, School of Medicine, Pontificia Universidad Católica de Chile, Santiago 8330077, Chile; Center for Translational Research in Endocrinology (CETREN-UC), Pontificia Universidad Católica de Chile, Santiago 8330074, Chile; Department of Endocrinology, School of Medicine, Pontificia Universidad Católica de Chile, Santiago 8330077, Chile; Center for Translational Research in Endocrinology (CETREN-UC), Pontificia Universidad Católica de Chile, Santiago 8330074, Chile; Department of Endocrinology, School of Medicine, Pontificia Universidad Católica de Chile, Santiago 8330077, Chile

**Keywords:** obesity, metabolic syndrome, adipocyte-derived extracellular vesicles (AdEVs)

## Abstract

Obesity is a major public health issue that is associated with metabolic diseases including diabetes mellitus type 2 and metabolic syndrome. This pathology leads to detrimental cardiovascular health and secondary effects, such as lipotoxicity, inflammation, and oxidative stress. Recently, extracellular vesicles (EVs) have been highlighted as novel players participating in human physiology and pathophysiology. In obesity, adipose tissue is related to the active shedding of adipocyte-derived extracellular vesicles (AdEVs). The current review explores and highlights the role of AdEVs and their cargo in obesity and metabolic syndrome. AdEVs are proposed to play an important role in obesity and its comorbidities. AdEVs are biological nanoparticles mainly shed by visceral and subcutaneous adipose tissue, acting in physiological and pathophysiological conditions, and also carrying different cargo biomolecules, such as RNA, microRNA (miRNA), proteins, and lipids, among others. RNA and miRNA have local and systemic effects affecting gene expression in target cell types via paracrine and endocrine actions. State of the art analyses identified some miRNAs, such as miR-222, miR-23b, miR-4429, miR-148b, and miR-4269, that could potentially affect cell pathways involved in obesity-related comorbidities, such as chronic inflammation and fibrosis. Similarly, AdEVs-proteins (RBP4, perilipin-A, FABP, mimecan, TGFBI) and AdEVs-lipids (sphingolipids) have been linked to the obesity pathophysiology. The current knowledge about AdEVs along with further research would support and reveal novel pathways, potential biomarkers, and therapeutic options in obesity.

Obesity is a condition characterized by an excessive accumulation of body fat due to genetic, socioeconomic, and environmental factors. First, some genes can influence the fat stores, metabolization of nutrients, and neuroendocrine signals, such as leptin, *MCR4*, *GLP1R*, and *FTO*, among others [1, 2]. Second, certain socioeconomic factors such as education, poor quality of food, and unhealthy lifestyles, may be linked to obesity. Third, there are environmental factors affecting obesity progression, such as the high-fat/sucrose diet, sedentarism, medications [3], and exposure to endocrine-disrupting chemicals (ie, pollutants) [4]. Obesity rates have increased worldwide, and as reported by the World Health Organization, more than 1.9 billion adults worldwide are classified as overweight (body mass index [BMI] ≥ 25 kg/m^2^), and one-third of those adults, 650 million, are obese (BMI ≥ 30 kg/m^2^). Obesity and excess body fat are major public health issues associated with increased incidence of cardiovascular disease, diabetes, and cancer [5].

Obesity is the most significant risk factor for the development of insulin resistance. Insulin resistance is caused by lipotoxicity produced by an abnormal accumulation of free fatty acids (FFAs) and triglycerides in peripheral tissues such as skeletal muscle and the liver in the pancreatic islets. Lipotoxicity and lipoapoptosis are linked to the generation of reactive oxygen species, resulting in cell apoptosis, chronic inflammation, fibrosis, and organic dysfunction [6]. The accumulation of lipids in pancreatic islets causes the proliferation of β-cells, which increases insulin secretion and maintains glucose homeostasis. However, an impaired compensatory mechanism leads to type 2 diabetes mellitus (T2D) and additionally metabolic syndrome (MetS) [7].

##  

### Obesity and Adipocyte Tissue Dysfunction

Obesity is a complex multifaceted chronic disease characterized by an abnormal and excessive accumulation of adipose tissue (AT) [8]. AT is the main source of energy in the body. Although adipocytes are the main building blocks of ATs [9], ATs are composed of other cells that form the stroma-vascular fraction, such as preadipocytes, macrophages, monocytes, stem cells, and endothelial cells, among others, all of which participate in AT plasticity. In mammals, ATs are categorized into 2 main types: white adipose tissue (WAT) and brown adipose tissue (BAT) [8, 10]. WAT is distributed throughout the body and represents approximately 10% to 15% of the body weight of a healthy subject and is the most important energy source in the body; moreover, it is mobilized according to the body's needs [11]. WAT includes subcutaneous adipose tissue (SAT) and visceral adipose tissue (VAT). SAT stores over 80% of total body fat, is under the skin, and is mostly distributed in the abdominal and gluteofemoral zones (Fig. 1). The VAT, located inside the abdominal cavity, stores 5% to 20% of body fat and it includes various adipose depots, such as mesenteric, epididymal white adipose tissue (EWAT), and perirenal depots [12]. The extension of both stores contributes to obesity. However, the development of metabolic diseases and cardiometabolic risk is mainly related to the increased abundance and activity of VAT [13]. This increased activity is related to the VAT secretome, which includes adipokines, proteins involved in adipose tissue inflammation, and novel adipocyte-derived extracellular vesicles (AdEVs) [14, 15].

**Figure 1.**
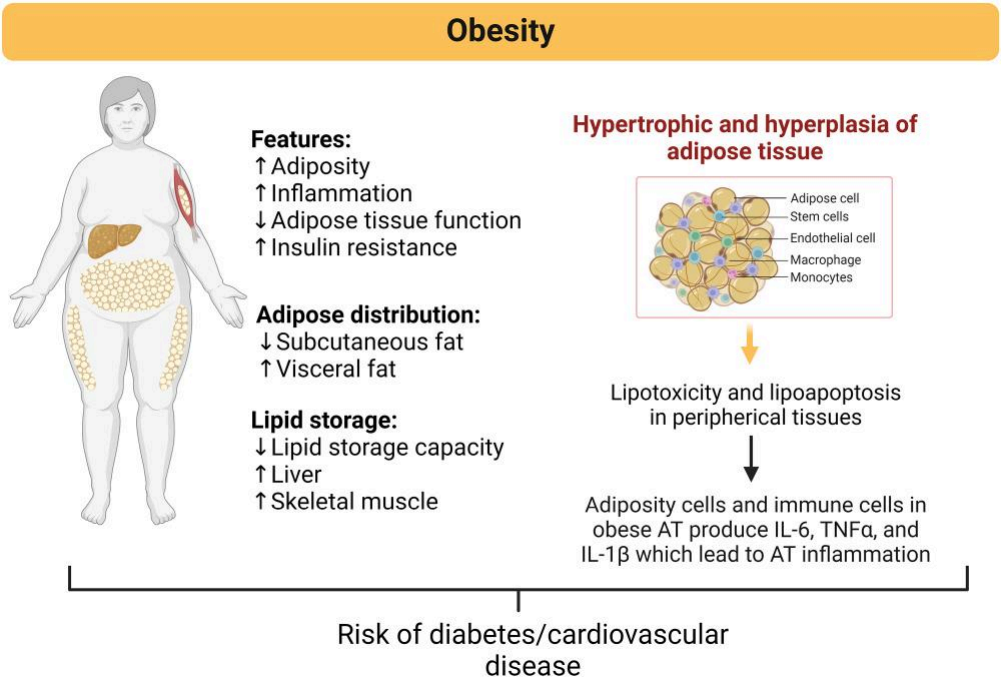
Adipose tissue features and distribution, and associated damage mechanisms underlying the pathophysiology of obesity. Adipose tissue undergoes several changes in its composition, distribution, and function that increase the risk of developing diabetes, cardiovascular complications, and other metabolic diseases. Unhealthy expansion of adipose tissue during obesity is mainly mediated by hyperplasia and hypertrophy of adipocytes, increasing adipose tissue inflammation driven by activation of proinflammatory adipokines and cytokines, such as IL-6 and TNF-alpha. Created with BioRender.com.

Communication of ATs with other key metabolic tissues is achieved through dense vascularization and innervation [16]. At the cellular level, energy is stored by lipids, namely, triacylglycerols, which are produced by adipocytes as large lipid droplets that ﬁll the cytoplasm and expand cells and tissue [13]. In obesity, AT becomes hypertrophic (increase in the size of existing adipocytes) and hyperplastic (forming new adipocytes through the differentiation of resident precursors known as preadipocytes). Differentiated adipocytes have remarkable hypertrophic potential and can increase in size to several hundred micrometers in diameter [17]. This increases mechanical stress due to contact with neighboring cells and increases hypoxia because of the limited diffusion of oxygen, which contributes to adipose tissue inflammation [18]. Larger adipocytes have been found to correlate with an increased proinflammatory adipokine profile and greater collagen deposition and fibrosis in adipose depots [17], contributing to a chronically stressed microenvironment in AT. Moreover, excess toxic lipid species, such as FFAs, which accumulate ectopically in different organs, generate a harmful effect known as lipotoxicity [19].

WAT also has endocrine functions and secretes many peptides, hormones, and steroids, all of which play homeostatic roles. This chronic, unresolved inflammatory state has detrimental effects on various organs and is supported by several metabolites released from adipose tissue, mainly from visceral WAT; these metabolites include peptides and adipokines (such as angiotensinogen, tumor necrosis factor-alpha [TNF-α], interleukin-6 [IL-6], fatty acid–binding protein 4 [FABP4], chemerin, adiponectin, and leptin), steroidal hormones (such as cortisol) and fatty acids (such as FFAs, diacylglycerol, and ceramides). Excess stored fat generates hypertrophy and hyperplasia of adipocytes with the consequent attraction of inflammatory cytokines and adipokines and infiltration of proinflammatory immune cells, causing low-grade systemic inflammation [20] (Fig. 1).

In addition to the mechanical and metabolic changes performed by adipocytes, AdEVs have recently emerged as new key actors with metabolic and communicative roles in AT due to their striking capacity to convey messages between cells.

### Adipocyte-Derived Extracellular Vesicles and Communication With Other Tissues and Organs

Adipocyte-derived extracellular vesicles (AdEVs) are proposed to play an important role in obesity and its comorbidities [9]. EVs are a diverse group of nanoparticles released or shed by different types of tissues and cells and act as intercellular messengers [21]. EVs are composed of a bilayered lipid membrane that encloses cytoplasmic soluble elements and are typically classified according to their biogenesis; these vesicles are subdivided into different subgroups: exosomes (30-150 nm), microvesicles (150-500 nm), and apoptotic bodies (0.5-5 μm) [22]. Exosomes, also known as small EVs (sEVs), are generated through the inward budding process of the late endosome membrane, forming the multivesicular body (MVB), which contains intraluminal vesicles [23].

Recent studies using high-fat diet–induced obese or genetically modified rodent models have shown an increase in the production of AdEVs [24-26]. In human patients with obesity, the levels of circulating EVs are significantly elevated compared to those in lean subjects, according to different studies, suggesting that extracellular vesicles released by the increased amount of adipose tissue from obese individuals could substantially contribute to these phenomena [15, 24, 27]. Similarly, human and animal *ex vivo* and *in vitro* studies have shown that the AdEVs profile is altered in obese patients with T2D [28, 29].

### Extracellular Vesicles and AdEVs as Biovectors

Most recent EV- or exosome-related studies have focused on the role of EVs in intercellular communication and signaling [23], and also potential utility as a biomarker in human pathology associated to the EV-cargo [30-32]. EVs are stable and transported in different biofluids, such as blood or urine, suggesting that they are interesting long-distance communication biovectors. The EV content consists of a specific subset of bioactive molecules, including nucleic acids, proteins, and lipids, among others, some of which may reflect the content of their parent cell. Nevertheless, some proteins are common to all exosomes regardless of their cellular origin, since they are linked to the metabolic pathway of exosomes generally associated with endosomes, the plasma membrane, and the cytosol [33]. Recently, there is interest in related proteins that have become useful EV markers. All exosomes contain membrane-bound molecules that allow them to be recognized by target cells. Once they attach to recipient cells, exosomes can induce modifications to different biological pathways through various mechanisms: via receptor-ligand interactions, via exosome internalization (by endocytosis and/or phagocytosis), or via exosome fusion with the plasma membrane of the target cell, delivering their contents to the cell cytosol [34]. AdEVs participate in intercellular and interorgan communication and regulate metabolic processes, such as energy flux, the immune system, and the pathophysiology of metabolic diseases [24].

### RNA and Noncoding RNAs as Specialized EV Cargos in AdEVs

EVs contain cellular RNA and noncoding RNA (ncRNA), especially microRNAs (miRNAs or miRs), which have the potential to deliver their miRNA cargo to recipient cells to affect the stability of individual mRNAs and the cell transcriptome. ncRNAs include a wide range of RNA families, such as those involved in the translation and splicing of messenger RNA (mRNA) as well as those associated with the modification of ribosomal RNA [13]. ncRNAs also play essential roles in multiple biological functions and are involved in metabolic processes [11]. Based on the size of their sequences, ncRNAs can be divided into short (∼20 to 200 nucleotides) and long ncRNAs (200 to ∼100 000 nucleotides) [16, 35]. miRNAs are a class of short noncoding RNAs (∼22 nucleotides) that are highly conserved and engaged in the post-transcriptional control of gene expression in multicellular organisms [10]. Its deregulation is associated with many human diseases, ranging from metabolic and inflammatory diseases to malignancies [12]. According to some studies suggesting an association between AdEVs and obesity-associated metabolic diseases, the composition of EVs released by adipose tissue in individuals with obesity-related comorbidities has been poorly studied. Recent reports suggest that miRNAs in AdEVs may play a more important role in circulation similar to proteins such as leptin, adiponectin, or FABP4, since exosomal miRNAs may also act as endocrine effectors to regulate metabolic homeostasis *in vivo* [36, 37]. Most of these studies are focused on adipose-derived exosomal miRNAs due to their ability to have far-reaching systemic effects, affecting for example proliferation, immune response, metabolic reprogramming, among others [38, 39]. In this way, AdEV-miRNAs could be used also as both biomarkers and molecular tools to improve research diagnostic and therapeutic efforts aimed at reducing obesity and associated mortality due to metabolic diseases [38].

### AdEV-miRNAs and Obesity and Its Comorbidities

Several studies have shown that AdEV-miRNA interactions change significantly during obesity and comorbidity development and may serve as biomarkers for disease diagnosis [36, 37]. We present a series of studies on exosomal miRNAs associated with obesity and metabolic diseases.

#### miR-222 and its association with insulin resistance

In 2020, Li et al highlighted that the adipocyte tissue–derived exosomal miRNA miR-222 promotes obesity-associated insulin resistance affecting the liver and skeletal muscle of high-fat diet–fed obese mice by suppressing the expression of insulin receptor substrate 1 (IRS-1), an intracellular signaling adapter protein that mediates many key metabolic signals initiated by insulin and its receptors and is the main substrate of insulin-like growth factor 1 (IGFR1) [39, 40]. Hence, impaired IRS-1 activity causes metabolic complications; for example, mice without IRS-1 (IRS-1-KO mice) exhibit insulin resistance [41]. Li et al suggested that miR-222 is a potential target for treating obesity-induced MetS and T2D [39].

#### miR-23b, miR-4429, miR-148b, miR-4269 and the inflammatory and fibrotic activities

Other important discoveries involving adipocyte-derived exosomal miRNAs and obesity have been made in recent years. In 2015, Ferrante and her group compared human obese vs lean adipocyte visceral exosomal miRNAs. Their study revealed that miR-23b, miR-4429, miR-148b, and miR-4269 are adipocyte-derived exosomal miRNAs that are differentially expressed between obese and lean donors, probably by altering transforming growth factor beta (TGF-β) and Wnt/β-catenin signaling [42]. This interaction seems to be important in the development and progression of inflammatory and fibrotic activities, since TGF-β signaling is implicated in fibrosis [43]. Taken together, these results suggest that the regulation of these pathways through these miRNAs could be used as a therapeutic approach for some obesity-related comorbidities, such as chronic inflammation and fibrosis [42].

#### miR-99b and the insulin-independent glucose uptake

Thomou et al also studied the role of circulating miRNAs derived from adipocyte tissue in both rodents and humans [37]. They found that adipocyte-derived exosomal miRNA-99b reduced hepatic fibroblast growth factor 21 (*FGF21*) mRNA levels *in vivo,* which was associated with a reduction in glucose tolerance. *FGF21* has been linked also to insulin-independent glucose uptake through the regulation of glucose transporter 1 (GLUT1) expression [44]. In that study, Thomou et al concluded that adipocyte-derived exosomal miRNAs may have far-reaching effects on multiple organs [37]; therefore, they could be used as therapeutic agents for metabolism regulation.

#### miR-155, miR-29a, miR-486, and miR-215-5p in glucose tolerance and diabetes nephropathy

In 2017 and 2019, Ying et al and Liu et al focused their studies on adipocyte tissue macrophage (ATM)-derived exosomal miRNAs, revealing that miR-155 and miR-29a are among the miRNAs overexpressed in ATM exosomes in rodent models and that both play important roles in glucose tolerance and insulin activity, influencing rodent metabolism [45, 46]. Instead of working with adipocytes or ATM exosomes, Jin et al focused on adipocyte-derived stem cell (ADSC) exosomes and their impact on diabetic nephropathy [47]. In 2019, his group reported that ADSC-exosomes improved diabetic nephropathy symptoms by increasing the expression of miR-486, which led to the inhibition of the Smad1/mTOR (Mothers Against Decapentaplegic family 1/Mammalian target of rapamycin) signaling pathway in podocytes [47]. Smad1/mTOR signaling transduction is involved in autophagy dysfunction-mediated podocyte injury, and hyperactivation of mTOR in diabetic nephropathy plays a fundamental role in podocyte injury [47]. According to the finding of Jin et al, ADSC-exosomes are excellent candidates for treatment of diabetic nephropathy. Ying et al also studied the protective effects of ADSC-derived exosomal miR-215-5p on podocyte injury associated with high glucose conditions, a pathological process characterized by podocyte epithelial-mesenchymal transition, which can cause podocyte dysfunction and diabetic nephropathy in the long term [48]. In podocytes, one of the downstream genes of miR-215-5p was ZEB2, a key regulator of epithelial-mesenchymal transition, suggesting that ADSC-derived exosomal miR-215-5p might relieve podocyte injury by regulating ZEB2 expression and that ADSC-derived exosomal miRNAs have potential as therapeutic agents for obesity-related diabetic nephropathy and other kidney diseases [48].

#### miRNA-6869-5p and the renal renin-angiotensin system

Liu et al investigated the role of adipocyte-derived exosomal miRNAs in kidney diseases. These findings indicated that obese EVs induced renal injury and local renal renin-angiotensin-aldosterone system (RAAS) stimulation in human proximal renal tubular epithelial cells *in vitro*. They found that obese EVs mediated the transport of miR-6869-5p, which is upregulated in these subjects, and activates RAS and promotes the expression of KIM-1 and NGAL, both markers of renal tubule injury. Therfore miR-6869-5p is a potential therapeutic target in local RAAS activation in obesity-associated kidney disease [49].

### AdEV Proteins and Obesity and Its Comorbidities

The state of the art identifies the associations of AdEV proteins with obesity and comorbidities. This review revealed specific proteins associated with AdEVs, such as retinol binding protein 4 (RBP4), perilipin-A, and FABP4.

#### Retinol binding protein 4

Deng et al determined that AdEVs mediate the activation of macrophages and that RBP4, a protein enriched in obese AdEVs, induces the production of TNF-α and IL-6 in macrophages in a TLR4-dependent manner, which is correlated with insulin resistance development [26].

#### Perilipin-A

Eguchi et al proposed perilipin-A as a marker of human adipocyte-derived EVs in obesity and suggested that the same protein can be used as a biomarker of adipocyte health [27]. This protein is a specific adipocyte protein that coats lipid droplets and plays a central role in the regulation of lipolysis [50]. Moreover, its levels are markedly increased in obese EVs, and proteomic analysis failed to detect this protein in EVs from healthy individuals [27].

#### Fatty acid–binding protein 4, mimecan, and TGFBI as biomarkers in individuals with obesity

Recently, proteomic analyses revealed novel potential AdEV markers, including fatty acid–binding protein 4 (FABP4/aP2) and perilipin-1 [24, 51]. Additionally, other studies by Camino et al confirmed that FABP4 is an AdEV-specific obesity marker and that both mimecan and transforming growth factor beta induced (TGFBI) are biomarkers for obesity comorbidities. According to this study, mimecan could be used for tracking adiposity since it was upregulated in obese AdEVs (specifically in VAT vesicles), and TGFBI to monitor T2D in obese patients. Moreover, compared with those in obese and lean patients, mimecan was upregulated in EVs from obese patients with a history of T2D [15].

### AdEV Lipids in Obesity and Comorbidities

The state of the art identifies the associations of AdEV lipids with obesity and comorbidities. Some examples are described below.

#### Lipids and sphingolipids

In 2023, Blandin et al used liquid chromatography–tandem mass spectrometry (LC-MS/MS) to identify a mouse AdEV-lipid signature in both healthy and obese mice. The present study revealed that the plasma of high-fat diet–fed obese AdEVs has a specific lipid fingerprint similar to that of VAT and plasma lipids and that includes increased lipids, such as phosphatidylcholine (PC), lysoPC species, sphingomyelin, ceramide, cholesterol esters, and free cholesterol, compared with their lean controls [52]. Crewe et al showed that AdEVs, which are associated with metabolic disease, were enriched in sphingolipids, a class of lipids that can be potent second messengers in pathological states such as T2D and could function as a marker of nutrient stress responses [53].

In summary, AdEVs and their cargo, such as miRNA and proteins, could exert different effects on other metabolic tissues (ie, liver, muscle, kidneys, and pancreas), which are associated with obesity-associated comorbidities (Fig. 2).

**Figure 2.**
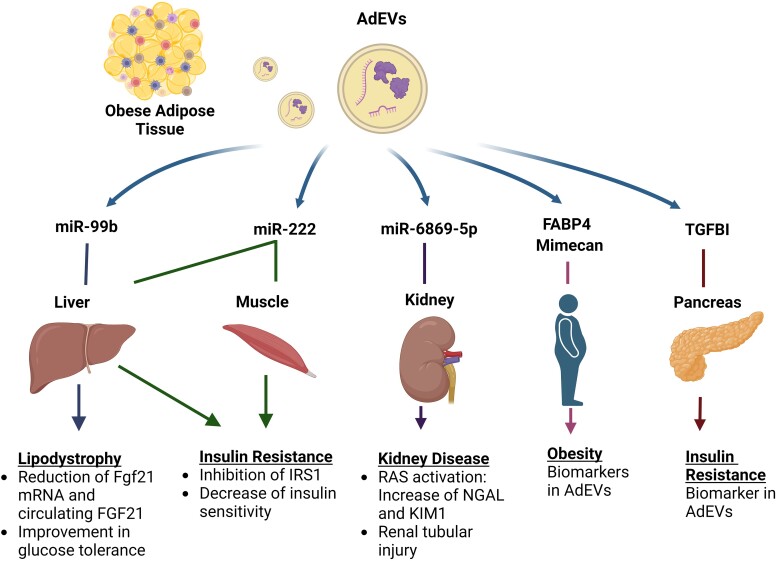
AdEVs and AdEV-cargos are associated with obesity-associated comorbidities by affecting other key metabolic tissues. Adipose-derived extracellular vesicles (AdEVs) and their cargo as RNA and proteins are a source of obesity biomarkers and also exert different effects on other metabolic tissues (ie, liver, muscle, kidneys, and pancreas), which support the obesity-associated comorbidities.

Abbreviations: AdEVs, adipocyte-derived extracellular vesicles; FABP4, fatty acid–binding protein 4; FGF21, fibroblast growth factor 21; IRS1, insulin receptor substrate 1; KIM-1, kidney injury molecule-1; NGAL, neutrophil gelatinase-associated lipocalin; RAS, renin-angiotensin system; TGFBI, transforming growth factor beta 1. Created with BioRender.com.

### AdEVs and AdEV-Cargos as Potential Diagnostic Biomarkers and Therapeutic Biomolecules in Obesity and Metabolic Syndrome

The ability of extracellular vesicles to incorporate, transport, and deliver different types of biomolecular cargo throughout the body has generated great interest in the scientific and clinical fields [54-56]. EV-mediated communication inspires the possibility of developing new strategies or technologies focused on the diagnosis and/or treatment of different metabolic diseases of global importance. In Table 1, we show the miRNAs, proteins, and lipids in AdEVs with biotherapeutic potential.

**Table 1.** Association of AdEVs with metabolic diseases and potential biomedical applications

	Name	Change	Species	Disease	Mechanism of action	Potential application	Reference
*Adipocyte-derived exosomal noncoding RNAs*							
miRNA	miR-23b	Upregulation	Human	Obesity, Metabolic syndrome	TGF-β and Wnt/β-catenin signaling pathways	Biomarker, Therapeutical	[42]
miRNA	miR-148b	Downregulation	Human	Obesity, Metabolic syndrome	TGF-β and Wnt/β-catenin signaling pathways	Biomarker, Therapeutical	[42]
miRNA	miR-4269	Downregulation	Human	Obesity, Metabolic syndrome	TGF-β and Wnt/β-catenin signaling pathways	Biomarker, Therapeutical	[42]
miRNA	miR-4429	Upregulation	Human	Obesity, Metabolic syndrome	TGF-β and Wnt/β-catenin signaling pathways	Biomarker, Therapeutical	[42]
miRNA	miR-99b	Upregulation	Human, Mouse	Lipodystrophy, Insulin resistance	Lowering *Fgf21* mRNA levels in the liver	Biomarker, Therapeutical	[37]
miRNA	miR-155	Upregulation	Mouse	Insulin activity	Downregulation of PPARg expression	Therapy for metabolic regulation	[45]
miRNA	miR-486	Upregulation	Mouse	Diabetic nephropathy	Suppression of Smad1/mTOR signaling pathway in podocytes	Therapeutical	[47]
miRNA	miR29a	Upregulation	Mouse	Insulin resistance	Targeting PPAR-δ	Therapeutical for T2D and metabolism regulation, 29	[46]
miRNA	miR-215-5p	Upregulation	Mouse	Kidney disease	Possibly through inhibition of ZEB2 transcription	Therapeutical for podocyte dysfunction and diabetic nephropathy	[48]
miRNA	miR-222	Upregulation	Mouse	Insulin resistance	Suppression of IRS-1 protein expression	Therapeutical for T2D and obesity-induced metabolic syndrome	[39]
miRNA	miR-6869-5p	Upregulation	Human Mouse	Obesity, Renal tubule injury	Unclear	Therapeutic for local RAAS activation in obesity-associated kidney disease	[49]
lncRNA	lncRNA-H19	Upregulation	Human	Diabetic foot ulcer	PTEN-mediated PI3K/AKT signaling pathway	Treatment for diabetic foot ulcer	[57]
circRNA	circ_0000250	Upregulation	Mouse	Diabetes	miR-128-3p/SIRT1-mediated autophagy	Treatment for diabetic ulcers	[67]
*Adipocyte-derived exosomal proteins*							
Protein	RBP4	Upregulation	Mouse	Insulin resistance	TLR4/TRIF pathway	Inmune, Therapeutical	[26]
Protein	Perilipin-A	Upregulation	Human, Mouse	Obesity, Metabolic syndrome	Regulation of lipolysis	Diagnosis, a biomarker of metabolic health	[27]
Protein	Sonic Hedgehog	Upregulation	Mouse (3T3 cells)	Insulin resistance	Macrophage polarization via Ptch and PI3K pathways	Therapeutical	[69]
Protein	FABP4	Upregulation	Human, Mouse	Obesity, Metabolic syndrome	—	Biomarker for obesity	[15]
Protein	Mimecan	Upregulation	Human	Obesity adiposity	—	Tracking adiposity on obese patient	[15]
Protein	TGFBI	Upregulation	Human	Obesity-induced insulin resistance	—	Monitoring T2D status in obese patients	[15]
*Adipocyte-derived exosomal lipids*							
Lipids	Sphingolipids	Upincorporation	Mouse	Obesity-induced insulin resistance	Second messengers for stress-related pathways	Marker for nutrient stress responses	[53]

Abbreviations: AdEV, adipocyte-derived extracellular vesicle; circRNA, circular noncoding RNA; FABP4, fatty acid–binding protein 4; IRS-1, insulin receptor substrate 1; lncRNA, long noncoding RNA; miRNA, microRNA; PPAR, peroxisome proliferator-activated receptor; RAAS, renin-angiotensin-aldosterone system; T2D, type 2 diabetes; TGF-β, transforming growth factor beta; TGFBI, transforming growth factor beta induced protein

An example of this type of RNA is an exosomal ncRNA, named *long noncoding RNA* (lncRNA), which has also been studied for its role in the physiopathology of obesity and its comorbidities. In 2020, Li et al documented that the mesenchymal stem cell (MSC)-derived exosomal lncRNA H19 stimulates wound healing in diabetic foot ulcers through the upregulation of PTEN by impairing microRNA-152-3p [57]. The PTEN-mediated PI3K/AKT signaling pathway leads to an increase in proliferation and migration and a decrease in fibroblast apoptosis, thereby accelerating the wound healing process.

Although there are no direct applications of AdEVs that support their use for the treatment of obesity or obesity comorbidities, several publications have evaluated the healing and/or regenerative properties of AdEVs. Since preadipocytes are recognized as sources of mesenchymal stem/stromal cells (MSCs), also known as adipocyte-derived stem cells [58], their isolated EVs are being studied for their beneficial properties [5, 59].

Previous studies have shown that MSC-derived extracellular vesicles (MSC-EVs) have therapeutic effects on tissue repair through their ability to exert antiapoptotic, anti-inflammatory, and antioxidant effects [56, 60]. MSC-EVs can enhance the proliferation and reduce the apoptosis of epithelial cells in kidney disease, hepatocytes in liver disease, and cardiomyocytes in heart disease, apparently through the delivery of RNA or growth factors [60]. Moreover, MSC-EVs isolated from adipocyte tissue (ADSC-EVs) have been shown to decrease inflammatory, fibrotic, and apoptotic pathway activity while promoting anti-inflammatory, antioxidant, prosurvival, and angiogenic signaling [58, 61-66].

Similarly, Shi et al verified that mmu_circ_0000250, an exosomal circular noncoding RNA (circRNA), enhances the therapeutic effects of ADSC-exosomes, promoting wound healing in diabetes by decreasing miR-128-3p and subsequently enhancing SIRT1 expression [67]. miR-128-3p can promote inflammatory responses and inhibit SIRT1 expression; therefore, its binding to mmu_circ_0000250 not only promotes SIRT1 expression but also reduces the inflammatory microenvironment [67]. On the other hand, an increasing number of studies suggest that SIRT1 promotes autophagy, a process that has a protective effect on cutaneous wound healing, so an increase in SIRT1 expression can improve wound healing [68]. Taken together, these findings indicate that ADSC-EXO-derived mmu_circ_0000250 promoted wound healing in diabetic mice by inducing miR-128-3p/SIRT1-mediated autophagy [67].

Although there are several potential applications of EVs in the clinical field, suitable and reliable procedures for the generation, isolation, and analysis of extracellular vesicles that meet MISEV guidelines and, eventually, international regulatory agency requirements, including those of the Food and Drug Administration (FDA), are needed to use EVs and EV cargos as biomarkers, vaccines, drug delivery devices, or therapeutic tools [55].

## Conclusion

The increase of adipose tissue in obesity is attributed to the formation of new adipocytes through the differentiation of precursors known as preadipocytes. Differentiated adipocytes have a marked potential for hypertrophy and increased inflammatory activity since they release proinflammatory cytokines (IL-1B, IL-6, and TNF-α), which impair metabolic health and trigger oxidative stress associated with lipotoxicity. These deleterious effects lead to insulin resistance, which can progress to diabetes mellitus and metabolic syndrome. Furthermore, these cellular and metabolic effects can be communicated through AdEVs to all tissues and organs of the human body through circulation and biofluids. AdEV-mediated communication with metabolic tissues may offer important insights into these adipocyte-derived EVs and EV cargos in the regulation of metabolic functions during disease and, secondarily, also offer potential diagnostic and therapeutic opportunities.

## Data Availability

Data sharing is not applicable to this article because no datasets were generated or analyzed during the study.

## Abbreviations

AdEV: adipocyte-derived extracellular vesicle
ADSC: adipocyte-derived stem cell
AT: adipose tissue
ATM: adipocyte tissue macrophage
BMI: body mass index
EV: extracellular vesicle
FABP4: fatty acid–binding protein 4
FFA: free fatty acids
IL-: interleukin
IRS-1: insulin receptor substrate 1
lncRNA: long noncoding RNA
MetS: metabolic syndrome
miRNA: microRNA
mRNA: messenger RNA
MSC: mesenchymal stem cell
ncRNA: noncoding RNA
RAAS: renin-angiotensin-aldosterone system
SAT: subcutaneous adipose tissue
T2D: type 2 diabetes
TGF-β: transforming growth factor beta
TNF-α: tumor necrosis factor-α
VAT: visceral adipose tissue
WAT: white adipose tissue
